# Assessment of the Palliative Prognostic Index in hospitalized oncologic patients treated by a palliative care team: impact of acute concomitant diseases

**DOI:** 10.18632/oncotarget.24826

**Published:** 2018-04-10

**Authors:** Carmen Palomar-Muñoz, Marina Martín-Zamorano, Amparo Mogollo, Susana Pascual-Pérez, Inmaculada Rodríguez-Morales, José-Antonio Girón-González

**Affiliations:** ^1^ Service of Internal Medicine, Palliative Care and Infectious Diseases, Hospital Universitario Puerta del Mar, Facultad de Medicina, Universidad de Cádiz, Instituto para la Investigación e Innovación en Ciencias Biomédicas de Cádiz (INiBICA), Cádiz, Spain

**Keywords:** cancer, palliative care, infectious diseases, survival, Palliative Prognostic Index

## Abstract

The differential prognostic accuracy of the Palliative Prognostic Index (PPI) in hospitalized oncologic patients treated by a palliative care team according to the presence or absence of acute concomitant diseases was analyzed. All patients (*n =* 322) hospitalized in a palliative unit of a university hospital were included in a 2-year prospective study. PPI was determined at the time of hospital admission and discharge. Patients were grouped into two categories according to the causes of hospitalization (presence and absence of acute concomitant diseases). Metastases, PPI punctuation, refractory symptoms, and the presence of acute concomitant diseases were analyzed as survival-related factors. The absence of acute concomitant diseases and a PPI calculated at admission >4 or >6 were related with survival at 3 and 6 weeks, respectively. After hospital discharge, the accuracy of PPI was lower, decreasing the positive predictive value from 84% (PPI calculated at the time of hospital admission) to 67% (PPI calculated at the time of discharge) for survival <6 weeks. In conclusion, the impact of acute concomitant diseases on survival should be considered in prediction models for patients receiving palliative care.

## INTRODUCTION

In patients receiving care in palliative care units (PCUs), information about prognosis is important to help patients set priorities and expectations for care and to assist clinicians in decision-making. Prognostic scores for patients in palliative care programs have been built and validated [1]. Specifically, the Palliative Performance Scale (PPS) score [2, 3] is a modification of the Karnofsky Performance Scale, in which ambulation, activity, self-care, intake, and level of consciousness are considered. The Palliative Prognostic Index (PPI) is the sum of the PPS and other clinical variables (oral intake, edema, resting dyspnea, and delirium) that are independently predictive of survival (4). The PPI can acceptably predict whether or not a patient will survive >3 or >6 weeks [4]. PPI has been tested by other authors [5–8].

The existence of concomitant diseases has not been taken into account in the building of prognostic scores, although acute concomitant diseases can impact prognostic scores. For example, a disseminated infection can decrease the level of consciousness, activity, and oral intake, or favor the presence of dyspnea and delirium [9], and thus modify the total score. The successful treatment of these concomitant diseases can also modify the prediction of survival.

Herein we assessed whether the survival of patients is not only dependent on the PPI score calculated at the time of hospital admission, but also on the presence or absence of acute concomitant diseases. Modifications to the prognostic ability of the PPI after hospital discharge of patients when symptoms have been controlled and acute concomitant diseases have been resolved were also assessed.

## RESULTS

Three hundred twenty-two patients were included. Of the 322 patients, 165 (51%) were hospitalized for acute concomitant diseases, as follows: infections, 109 cases (66%); acute hemorrhage, 16 cases (10%) [digestive, 9 cases; urinary, 7 cases]; cardiovascular acute syndromes, 16 cases (10%) [cardiac failure, 10 cases; cardiac arrhythmias, 4 cases; coronary insufficiency 2 cases]; pulmonary thromboembolism, 11 cases (7%); opioid-induced neurologic syndromes, 7 cases (4%); and miscellaneous, 6 cases (4%) [acute ischemic attack, 2 cases; epileptic crisis, 2 cases; and diuretic-induced hyponatremia or hypopotassemia, 2 cases]. At the discretion of the attending physician, all acute concomitant diseases were treated. Patients hospitalized without acute concomitant diseases (*n =* 157) [49%] presented as follows: refractory symptoms, 88 cases (56%); disease progression, 57 cases (36%); and caregiver’s or patient’s exhaustion, 12 cases (8%).

The characteristics of the 322 patients included in the study are shown in Table 1. There was no significant difference in the mean PPI score between the two groups of patients. Analyzing those factors contributing to the PPI, only a lower oral intake was significantly different between both groups (patients without vs. patients with acute concomitant diseases [PPS, 40 ± 14 vs. 36 ± 18, *p* = 0.578]); oral intake (preserved or reduced, 33% vs. 58%, p = 0.028); edema (38% vs. 43%, *p* = 0.427); dyspnea at rest (34% vs. 42%, *p* = 0.136); and delirium (38% vs. 43%, *p* = 0.427), respectively.

**Table 1:** Characteristics of the patients analyzed according to the presence or absence of acute concomitant diseases

Variable	Global (*n* = 322)	Patients without acute concomitant disease (*n* = 157)	Patients with acute concomitant disease (*n* = 165)	p*
Age (years)	71 ± 13	68 ± 14	73 ± 11	<0.001
Sex male (*n*, %)	196 (61)	89 (57)	107 (65)	0.139
Primary neoplasia (*n*, %)				
Respiratory	83 (26)	33 (21)	50 (30)	
Gastrointestinal, liver, bile duct, pancreas	115 (36)	63 (40)	52 (32)	
Genitourinary	57 (18)	28 (18)	29 (18)	
Breast	21 (7)	12 (8)	9 (5)	
Central nervous system	15 (5)	7 (4)	8 (5)	
Others	31 (10)	14 (9)	17 (10)	
Presence of metastasis (*n*, %)	249 (77)	127 (81)	122 (74)	0.145
Palliative Prognostic Index (Total score)	7.1 ± 3.4	7.0 ± 3.5	7.2 ± 3.3	0.521
Palliative Prognostic Index score				0.168
< 4.0	66 (21)	35 (22)	31 (19)	
4.1–6.0	75 (23)	42 (27)	33 (20)	
> 6.1	181 (56)	80 (51)	101 (61)	

^*^p patients with vs patients without acute concomitant diseases.

### Mortality of patients with or without concomitant diseases

During the hospitalization, there were 205 deaths. The mean length of hospital stay was 11 ± 9 days (patients with acute concomitant disease, 11 ± 10 days; patients without acute concomitant diseases, 10 ± 8 days, *p* = 0.863).

An analysis of factors associated to mortality is shown in Table 2. Linear regression analysis demonstrated that the presence of a PPI score ≥ 4.1 and the absence of concomitant diseases were independent factors associated with mortality (Table 2).

**Table 2:** Factors associated to mortality during hospitalization of patients

Variable	Surviving patients (*n* = 117)	Non surviving patients (*n* = 205)	*p* univariate	Multivariate analysis	
				Exp (B) (95% confidence interval)	*p*
Age (years)	72 ± 11	70 ± 13	0.057		
Sex male (n, %)	82 (70)	114 (56)	0.013		
Presence of metastasis	90 (77)	159 (78)	0.891		
Presence of acute concomitant diseases	73 (62)	92 (45)	0.003	0.38 (0.23–0.63)	<0.001
PPI score (Total score)	5 ± 3	8 ± 3	<0.001		
PPI score			<0.001		
<4.0	41 (35)	25 (12)		1	
4.1–6.0	34 (29)	41 (20)		3.25 (1.79–5.91)	<0.001
>6.1	42 (36)	139 (68)		6.50 (3.43–12.29)	<0.001

### Survival of patients with or without concomitant diseases from hospital admission

The median (interquartile range) survival time for the entire cohort of patients was 15 days (6–36 days). Patients without acute concomitant diseases had a survival of 14 days (6–30 days), whereas those with concomitant diseases showed a survival of 17 days (6–40 days), *p* = 0.210.

According to the PPI score, the survival of each group was as follows: A) patients with a PPI < 4.0, 38 days (13–76 days); B) patients with a PPI 4.1–6.0, 24 days (12–44 days); and C) patients with a PPI ≥ 6.1, 9 days (4–23 days); A vs. B, *p* = 0.097; A vs. C, *p* < 0.001; and B vs. C, *p* < 0.001.

**Figure 1:**
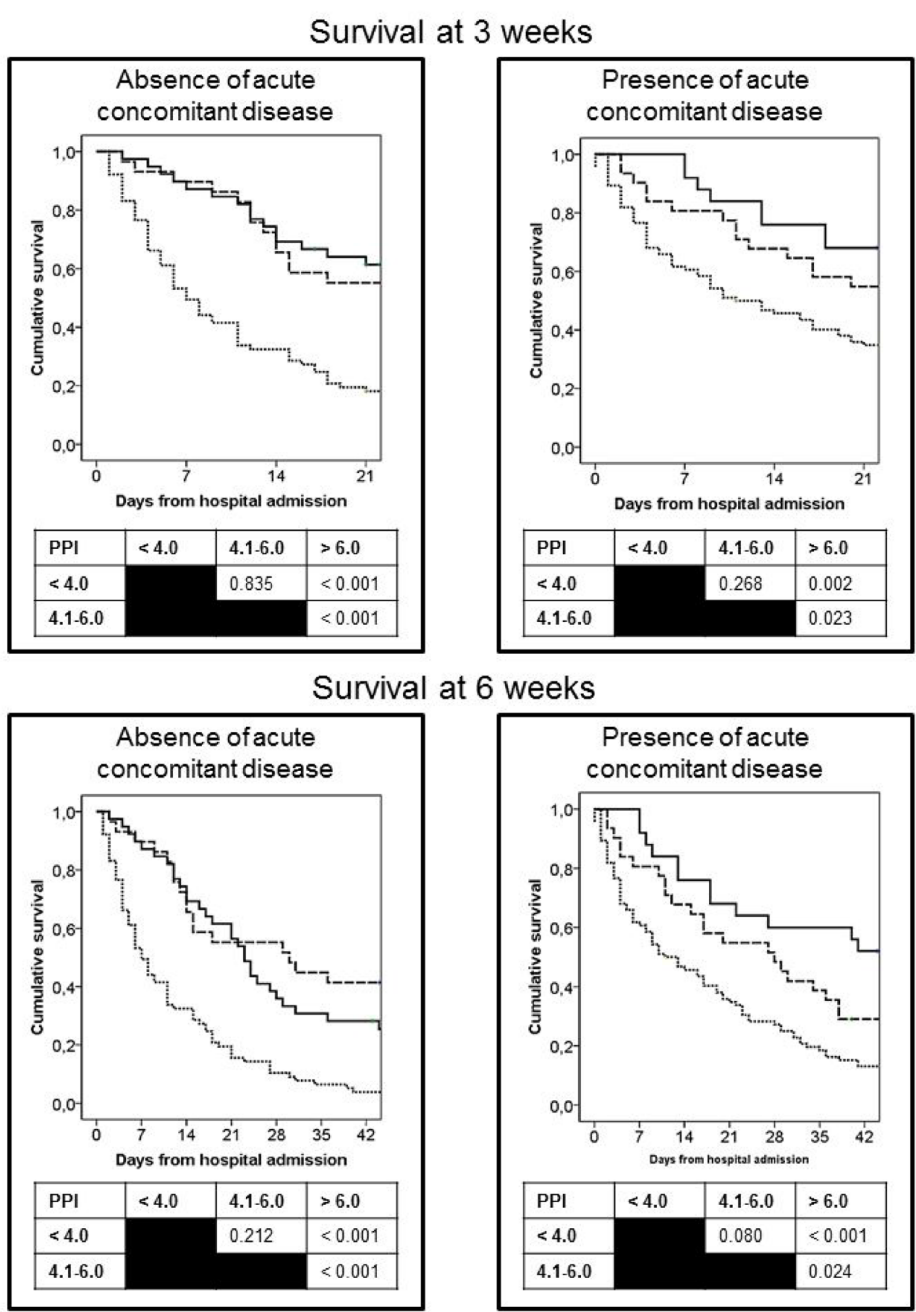
Curves of survival at 3 and 6 weeks of patients from hospital admission as a function of the PPI score Patients were grouped according the presence or absence of acute concomitant diseases. PPI score: <4 points (continuous line), 4.1–6.0 points (dashed line), >6.1 points (dotted line).

At 3 and 6 weeks, 143 (44%) and 73 (23%) patients, respectively, had survived. Survival at 3 and 6 weeks from the time of admission was analyzed according to the presence or absence of acute concomitant diseases and the PPI score. Logistic regression analysis of both variables showed that the absence of acute concomitant diseases and a PPI score ≥ 6.1 were independent factors associated with survival < 3 weeks. Absence of concomitant diseases and a PPI score ≥ 4.1 were independent factors associated with survival < 6 weeks (Table 3). Survival curves of patients, classified according to the presence or absence of acute concomitant diseases and to the PPI score, are shown in Figure 1.

**Table 3:** Survival at 3 and 6 weeks of studied patients

Variable		Patients surviving <3 weeks (*n* = 179)				Patients surviving <6 weeks (*n* = 249)			
		*N* (%)	*p* univariate	Multivariate analysis		*N* (%)	*p* univariate	Multivariate analysis	
				Exp (B) (95% confidence interval	*p*			Exp (B) (95% confidence interval	*p*
Acute concomitant diseases	Absent (*n* = 157)	94 (60)	0.145	1		130 (83)	0.024	1	
	Present (*n* = 165)	85 (52)		0.58 (0.36–0.93)	0.025	119 (72)		0.41 (0.22–0.73)	0.003
PPI score	A: ≤ 4.0 (*n* = 66)	21 (32)	A vs B: 0.295 A vs C: < 0.001 B vs C: < 0.001	1		33 (50)	A vs B: 0.005 A vs C: < 0.001 B vs C: 0.004	1	
	B: 4.1–6.0 (*n* = 75)	31 (41)		1.50 (0.74–3.01)	0.258	55 (73)		2.81 (1.37–5.77)	0.005
	C: ≥ 6.1 (*n* = 181)	127 (70)		5.45 (2.93–10.12)	< 0.001	161 (89)		9.41 (4.68 – 18.92)	<0.001

**Figure 2:**
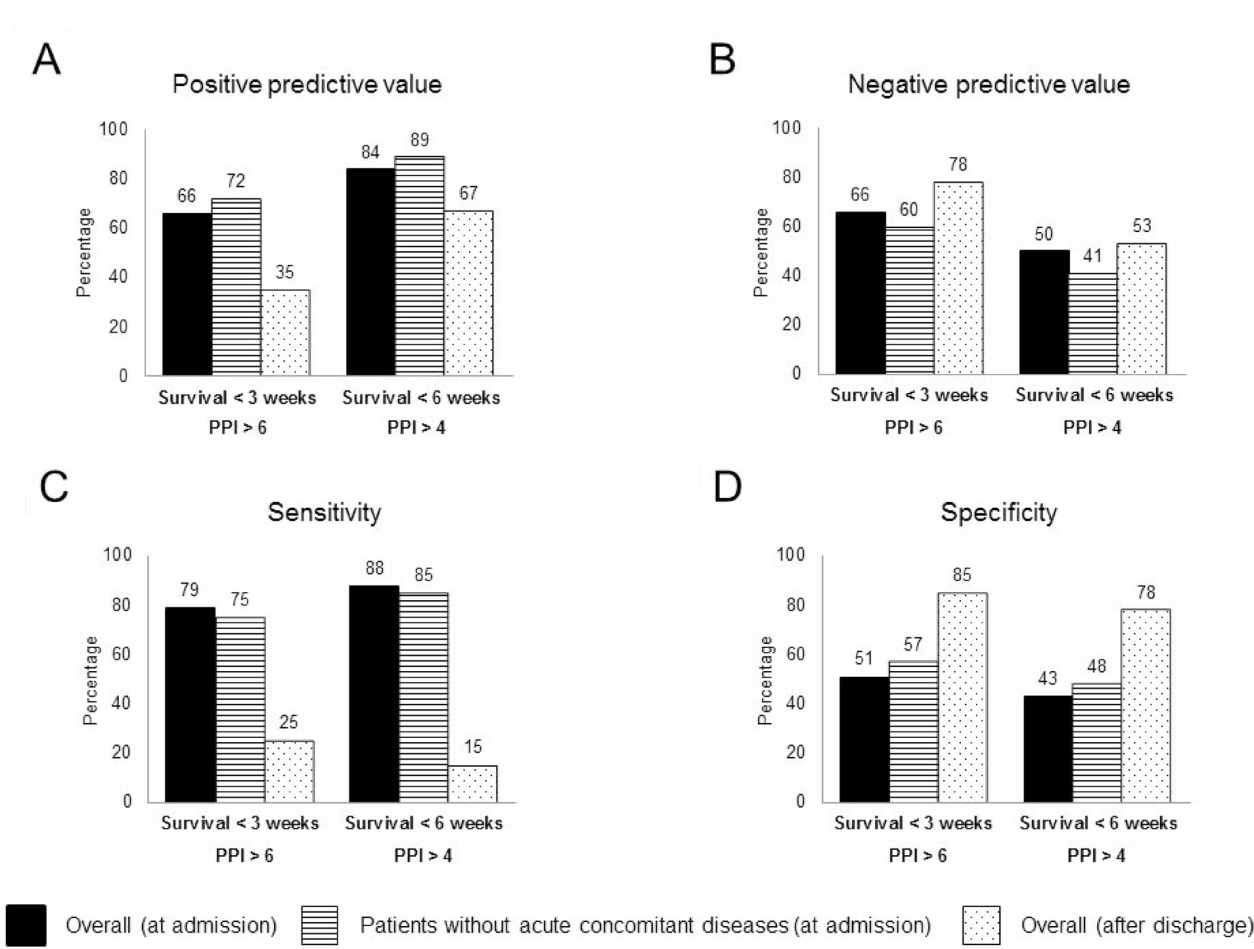
Accuracy of predictions using the PPI score at hospital admission (in the global cohort or only in patients without acute concomitant diseases) and after discharge ((**A**) Positive predictive value; (**B**) Negative predictive value; (**C**) Sensitivity; (**D**) Specificity).

Depending on the presence or absence of acute concomitant diseases and on the PPI calculated at the time of hospital admission, PPV, NPV, S, and E for survival < 3 weeks and < 6 weeks are shown in Figure 2A–2D.

### Survival after hospitalization

At 3 and 6 weeks, 76% (*n =* 89) and 46% (*n =* 54), respectively, of discharged patients (*n =* 117) had survived.

The mean PPI, calculated at the time of hospital discharge, of the 117 surviving patients was 3 ± 2 points (PPI < 4.0, *n* = 81, 70%; PPI = 4.1–6.0, *n* = 25, 21%; PPI > 6.0, *n* = 11, 9%). The median (interquartile range) survival of each group was as follows: patients with a PPI < 4.0, 38 days (17–75 days); patients with a PPI of 4.1–6.0, 31 days (18–110 days); patients with a PPI > 6.1, 24 days (15–56 days; *p* > 0.05 for each comparison).

According to the PPI calculated at the time of hospital discharge, PPV, NPV, S, and E of a PPI ≥ 6.1 for survival < 3 weeks and a PPI ≥ 4.1 for survival < 6 weeks are shown in Figure 2A–2D.

## DISCUSSION

The European Association for Palliative Care recommends the use of clinical tools, such as predictive scores to prognosticate life expectancy in advanced cancer patients [1]. Although there are several prognostic scores in Palliative Care [10], the Palliative Prognostic (PaP) Score, the Delirium-PaP score, and the PPI are the most frequently used [11, 12]. The accuracy at 21 days of follow-up of PaP score is 70–90%, 68–91% for Delirium-PaP score and 65–88% for PPI [11, 12]. The PaP and Delirium-PaP scores include total white blood count and lymphocyte percentage because these values can be modified by the presence of some concomitant diseases, such as infections, thus the PaP and Delirium-PaP scores were not considered suitable for the present study.

The PPI was built and internally validated in 1999 [4, 13]. The predictive value of the PPI varies among different studies [5, 6]. A possible reason is that the PPI is usually assessed only once, generally during the consultation with the palliative care specialist. When used only at the initial assessment, the PPI might be inappropriate as a prognostic tool because the PPI does not reflect the clinical course [14]. Prognosis may change based on the treatment response, development of acute oncologic complications (e.g., hypercalcemia and spinal cord compression), or competing co-morbidities (e.g., heart failure) [15]. Consequently, to be able to understand the results of the different studies, the time at which PPI was calculated is needed; however, only some studies [16] include this information.

We consider that the present study has contributed to assessment of the prognostic value of the PPI at two specific times (hospital admission [when symptoms are not controlled or a concomitant disease is present] and hospital discharge [symptoms are controlled and the patient is stable]).

The causes of hospital admission can be classified into two groups (not directly related to the cancer [concurrent diseases] and related to the cancer [either refractory symptoms, disease progression, or familiar or personal exhaustion]). This classification has been useful in the present work because it has been demonstrated that concurrent diseases modify the predictive ability of the PPI. Thus, analyzing survival < 3 weeks, the positive predictive value of the PPI ≥ 6.1 points is 66% if we analyze the overall cohort, but increases to 72% when only those patients with symptoms related with progression of the cancer are considered. Multivariate analyses demonstrated that in addition to the ability of the PPI to predict the prognosis, the presence of concurrent diseases impacts the probability of death in cancer patients. Several reasons could be accepted to explain these findings. First, concurrent diseases can modify the value of variables included in the PPI; thus, dyspnea or edema can be the consequence of cardiac failure or pneumonia; dyspnea can appear as a consequence of pulmonary thromboembolism [17], or delirium can occur because of the vasodilation and cerebral hypoxia in sepsis [9]. Second, the evolution of these patients with a treatable disease, even in palliative care, is better than the evolution of those who are attended by refractory symptoms or progression of the disease. Effectively, analyzing the mortality during hospitalization, it was observed that 92 of 165 patients (45%) with concurrent diseases died, whereas in those admitted for cancer-related causes the mortality was 72 % (113 of 157 admitted patients).

The issue of in-hospital mortality associated with palliative care has been previously addressed. Hui *et al.* [18], in a study involving 352 patients admitted to acute palliative care units, observed that the presence of acute symptomatic complications was associated with a higher risk of mortality; however, this study did not differentiate among those acute complications (bowel obstruction or perforation, cachexia, hemoptysis, hypercalcemia, or tamponade) probably related to cancer progression and those not related to cancer progression (such as heart failure, ischemic stroke, or pneumonia). This is the first study to analyze the influence of real concomitant diseases, not directly related with the neoplasia, on the prognostication provided by predictive scores. Findings of our work could explain the variability of PPI among different studies. Thus, for a survival < 3 weeks, the predictive positive value of a PPI > 6.1 ranges from 57 to 92% [4, 5, 7, 11, 12, 20–22].

After discharge, there was no significant difference in survival based on the PPI score. Moreover, the positive predictive value of the PPI calculated at hospital discharge decreased notably compared with the positive predictive value of the PPI at the time of hospital admission. This finding could be attributed to the poor prognosis related with a high symptom burden at the time of hospital admission [19]. In fact, the effective management of these symptoms by a palliative care team has been associated with improved survival in a controlled trial [20]. Other studies analyzing the survival of oncologic patients specifically in the home care setting have also detected a low accuracy for the PPI [21, 22].

In conclusion, the PPI, as a prognostic score in cancer palliative care, is dependent upon the moment at which the PPI has been calculated. Although the PPI retains a good predictive positive value at the time of hospital admission, it is mandatory to consider the possibility of acute concomitant diseases not related with the neoplasia, the treatment of which could modify symptoms and survival.

## PATIENTS AND METHODS

This observational, prospective, cohort study was conducted on patients consecutively admitted to the PCU of University Hospital Puerta del Mar (Cádiz, Spain) from December 2013 to December 2015. During the study period, all patients > 18 years of age referred to the PCU were considered eligible, and all were cancer cases. No patient was receiving palliative radiotherapy and/or chemotherapy.

The PCU includes a 14-bed inpatient unit in which this work was performed. Causes of admission of individuals under palliative care in this inpatient unit are as follows: 1) refractory symptoms/disease progression; 2) caregiver or patient exhaustion (physical or emotional); and 3) acute concomitant diseases.

### Study schedule

The following data were collected at the time of admission by the physician: age; sex; diagnoses of malignancy type; presence or absence of metastasis; and causes of admission and diagnosis of acute concomitant diseases, if any, and variables needed to calculate the PPI score (oral intake, presence or absence of edema, dyspnea at rest, delirium, and PPS). If patients were receiving total parenteral nutrition or had an enteral feeding tube, they were included in a “normal” oral intake category. Delirium was diagnosed at the time of admission using the criteria of the Diagnostic and Statistical Manual of Mental Disorders (Fifth Edition). For patients who had difficulty with verbal communication, a nurse specialist assessed their status using a proxy or caregiver response.

Patients were divided into good (score, 0–4.0), intermediate (score, 4.1–6.0), and poor (score, > 6.1) prognostic groups, according to the PPI score [5].

After treatment of hospitalized patients, all surviving individuals discharged were followed until death or study closure time (15 February 2016) by the mobile palliative care team.

Survival was analyzed considering two start points: 1) survival time from the first day of hospitalization to the day of death; and 2) survival time from the day of hospital discharge to the day of death.

### Statistical analysis

Qualitative variable results are shown as a number (percentage) and quantitative variables as a mean (± standard deviation) or as a median (interquartile range) depending on whether or not the data was normally distributed, as assessed by the Kolmogorov´ test.

To analyze the differential characteristics associated with mortality, qualitative variables were compared by the chi-square test with Fisher´s correction when appropriate, and quantitative variables by variance analysis or the Mann Whitney *U* test. Multivariate analysis of survival with a logistic regression model retained the variables significant at a *p* value < 0.05 after the univariate analysis.

For the calculation of survival from hospital admission, Kaplan-Meier survival curves were constructed for each of the two groups based on the presence or absence of acute concomitant diseases and for each of the three subgroups of the PPI. For the calculation of survival from hospital discharge, Kaplan-Meier survival curves were constructed for each of the three subgroups of the PPI.

Sensitivity (S), specificity (E), positive predictive value (PPV), and negative predictive value (NPV) of the PPI as predictors of survival of < 3 and 6 weeks were calculated.

### Ethics statement

Approval for the study was granted by the Ethics and Medical Research Committee at the University Hospital Puerta del Mar (Cadiz, Spain). All participants provided written informed consent.

### Availability of data and material

The datasets during and/or analysed during the current study are available from the corresponding author on reasonable request.
